# Cariogenicity features of Streptococcus mutans in presence of rubusoside

**DOI:** 10.1186/s12903-016-0212-1

**Published:** 2016-05-11

**Authors:** Jinpu Chu, Tieting Zhang, Kexin He

**Affiliations:** College of Stomatology, Zhengzhou University, Nanyang Road 169-10#, Zhengzhou, 453002 China; College of Stomatology, Guangxi Medical University, Shuangyong Road 10#, Nanning, 530021 China

**Keywords:** Rubusoside, Streptococcus mutans, Non-cariogenic, Sugar substitute

## Abstract

**Background:**

One promising way of reducing caries is by using sucrose substitutes in food. rubusoside is a prototype sweet substance isolated from the leaves of the plant *Rubrus suavissimus S. Lee.* (Rosaceae), and is rated sweeter than sucrose. The purpose of this study was to investigate the effects of rubusoside on *Streptococcus mutans* growth, acidogenicity, and adherence to glass *in vitro*.

**Methods:**

The effects of rubusoside on the growth and glass surface adhering of *Streptococcus mutans* were investigated by measuring the optical density of the culture at 540 nm with a spectrophotometer. Rubusoside influence on *Streptococcus mutans* acidogenicity was determined by measuring the pH of the culture. Sucrose, glucose, maltose, fructose and xylitol were designed to compare with rubusoside.

**Results:**

*S. mutans* growth in the rubusoside-treated group was significantly lower than that in the sucrose, glucose, maltose and fructose groups (*p* < 0.05) except for xylitol group (*p* > 0.05). Sucrose-treated *S. mutan*s exhibited the highest adherence to glass, and rubusoside-treated *S. mutan*s exhibited the lowest. *S. mutans* adherence to a glass surface and acidogenicity with sucrose were significantly reduced by rubusoside.

**Conclusions:**

Rubusoside may have some potential as a non-cariogenic, non-caloric sweetener.

## Background

Dental caries is a multifactorial and chronic bacterial disease that involves the destruction of tooth hard tissue structure. It is directly caused by the acid produced by oral bacteria fermentation of dietary carbohydrates in dental plaque [1]. Dental plaque represents a microbial ecosystem in which non-mutans bacteria (mainly non-mutans streptococci and Actinomyces) are the key microorganisms responsible for maintaining dynamic stability on the tooth surface (dynamic stability stage) [2, 3]. Subjects that frequently consume a considerable amount of fermentable carbohydrates, select for bacteria that ferment these carbohydrates and produce acids. This leads to more sugar fermentation and thus acid production, increasing the cariogenic bacteria even more. Acidogenic (acid-producing) and aciduric (acid-tolerating) bacteria such as the classic *Streptococcus mutans, Streptococcus sobrinus* and *Lactobacillus* spp., and the later discovered *Bifidobacterium* spp., lower the pH to levels at which enamel is demineralized, which can result in caries [4]. *S. mutans* is the major microbial etiological agent of dental caries, due to its ability to adhere to the tooth surface, by producing sticky extracellular polysaccharides from sucrose, and to ferment sucrose and other sugars to acids which attack the tooth enamel [5, 6]. There has been broad consensus that quantity and frequency of consumption of sucrose-containing foods is correlated with caries incidence [7, 8]^.^ Strict, long-term restriction of cariogenic sugars undoubtedly results in significant caries reduction. However, considering the human preference for sweet food items, restriction of cariogenic sugars without offering alternatives is impractical [9]. Therefore, especially for patients who are susceptible to dental caries, the use of non-cariogenic sugar substitutes should be considered.

**Fig. 1:**
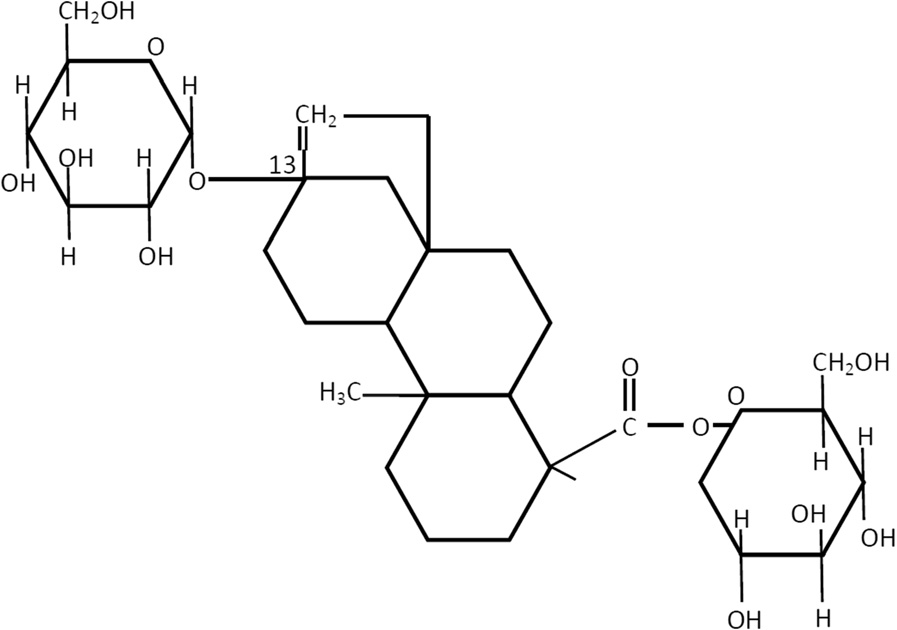
Chemical structures of rubusoside from Rubrus suavissimus *S. Lee*

Plants contain a number of highly sweet compounds, several of which are used commercially in one or more countries, particularly in Japan [10]. Rubusoside is a prototype sweet substance isolated from the leaves of the plant Rubrus suavissimus S. Lee. (Rosaceae), a natural non-toxic traditional Chinese medicine [11]. Historically, Rubrus suavissimus S. Lee leaves have been used by people in the Guangxi province (People’s Republic of China) as tea to drink and to prevent and cure diseases. Rubusoside, structurally characterized in the early 1980s, is a diterpene glycosides consisting mainly of steviol and dextrose (C32H50O13) [12], (Fig. 1) and was rated as sweeter than sucrose. Rubusoside is investidated to be a low toxicity, light side effect material. Liang et al studied the acute and chronic toxicity of Rubusoside. They found that Rubusoside had no toxicity on the development, hematology, function of the liver and kidney and histology in rats [13]. Rubusoside widely used as a natural sweetener for seasoning and additive in food industry [12]. The purpose of this study was to investigate the effects of Rubusoside on in vitro *S. mutans* cariogenic properties and to determine possibility to use as a non-cariogenic sugar substitute.

## Methods

### Saccharides

A highly purified preparation of rubusoside (98 % pure) was kindly provided by Guangxi Jinxiu Shengtang Pharmaceutical Co. Ltd. (China). Sucrose, glucose, maltose, fructose and xylitol were reagent-grade (Sigma, Saint Louis, MO, USA).

### Microorganisms

Stock cultures of S. mutans Ingbritt 1600 (serotype c) were maintained at 4 °C on brain-heart infusion agar slants. Bacterial identity was confirmed before and after testing previously described by Drucker and Green [14]. Washed cell-suspensions were prepared from 200 mL samples of brain-heart infusion broth, inoculated with 5 mL starter culture and incubated micro-aerophilically for 18 h at 37 °C. Cultures were re-incubated with 1 volume of fresh medium for 1 h, centrifuged at 3000 g for 15 min at 4 °C, and washed three times with cold, sterile, phosphate-buffered saline (PBS) and resuspended in PBS to an OD_540_ = 1.0 using a spectrophotometer (LAMBDA BIO 20, PerkinElmer, Massachusetts, USA), corresponding to ~5.0 × 10^8^ CFU/mL. Cell suspensions were used immediately.

### Test solutions and media

Test solutions consisted of Trypticase (2 %), NaCl (0.2 %), K_2_HPO_4_ (0.3 %), KH_2_PO_4_ (0.2 %), K_2_CO_3_ (0.1 %), MgSO_4_ (0.012 %), and MnSO_4_ (0.0015 %), An appropriate amount of yeast extract and the cysteine in distilled water which was used as the basic suspending fluid in all experiments [15].

The test solutions (50 mM of each carbohydrate) were sucrose, glucose, maltose, fructose, rubusoside, rubusoside + sucrose and xylitol. Sucrose and the basic suspending fluid were used as positive and negative control. Carbohydrate solutions of sweeteners in dH_2_O were sterilized by membrane filtration and the pH was adjusted to 7.4 with 10 mM NaOH.

### Saccharides influence on *S. mutans* growth

The original cell suspension (1 mL) was added to 15 mL of the various test solutions: the basic suspending fluid; or the basic suspending fluid include saccharides; sucrose, glucose, maltose, fructose, rubusoside, rubusoside + sucrose or xylitol, and incubated micro-aerophilically for 48 h at 37 °C. After, test suspensions were centrifuged at 3000 g for 15 min at 4 °C, and the cells were washed three times with cold, sterile, PBS and resuspended in PBS. The suspensions were homogenized by sonic oscillation. The optical density (OD _540_) of the aqueous phase was then read to compare the cell growth in the various media used. Experiments were performed in triplicate.

### Saccharides inhibition of *S. mutans* adherence to a glass surface

To assess the bacterial adherence of growing cells of S. mutans to a glass surface, the original cell suspension (1 mL) was added to 20 ml of the various test solutions: the basic suspending fluid; or the basic suspending fluid include saccharides; sucrose, glucose, maltose, fructose, rubusoside, rubusoside + sucrose or xylitol, in a glass test tube (15 × 150 mm) containing a glass rod (5 × 100 mm), and incubated micro-aerophilically for 48 h at 37 °C. After, the glass rod was carefully taken out from the culture, and the microbial deposits on the glass rod were washed for 10 s with dH_2_O to remove the cells that grew in close contact with the glass surface but did not actually adhere. The the glass rod was then suspended in 20 mL of water by vigorous vibration with a mixer, as adherent cells of strains can be removed by being mixed with a vortex blender. The suspensions were centrifuged at 3000 g for 15 min at 4 °C; and the cells were washed three times with cold, sterile PBS and resuspended in sterile PBS. The suspensions were homogenized by sonic oscillation. Optical density at OD_540_ of the suspensions was measured with a spectrophotometer. Experiments were performed in triplicate.

### Saccharides influence on *S. mutans* acidogenicity

For assaying pH reduction, the original cell suspension (1 mL) prepared was added to 15 ml of the various test solutions: the basic suspending fluid; or the basic suspending fluid include saccharides; sucrose, glucose, maltose, fructose, rubusoside, rubusoside + sucrose or xylitol, and incubated micro-aerophilically with shaking for 48 h at 37 °C. After, the pH of the solutions was measured via a pH-meter (MP230, Mettler-Toledo, Inc., Switzerland). Experiments were performed in triplicate.

### Statistical analysis

Data were analyzed using SPSS 17.0 software by ANOVA, followed by a post-hoc Newman–Keuls test with ɑ = 0.05.

## Result

### Saccharides influence on *S. mutans* growth

**Fig. 2:**
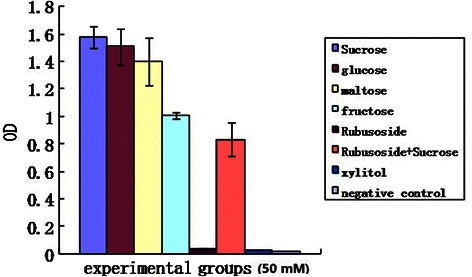
Effects of various experimental groups on the growth of *S. mutans*

The effect of the various Saccharide solutions on *S. mutans* growth is shown in Fig. 2. The rubusoside-treated group was significantly lower than that of the sucrose (positive control), glucose, maltose and fructose groups (*p* < 0.05). No significant differences in OD_540_ was observed when comparing the rubusoside-treated group to the xylitol group and negative control (*p* > 0.05). When rubusoside was incubated with *S. mutans* in the presence of sucrose, *S. mutans* growth was higher than that with rubusoside alone (*p* < 0.05), but significantly lower than in the positive control (sucrose) (*p* < 0.05).

### Saccharides inhibition of *S. mutans* adherence to a glass surface

**Fig. 3:**
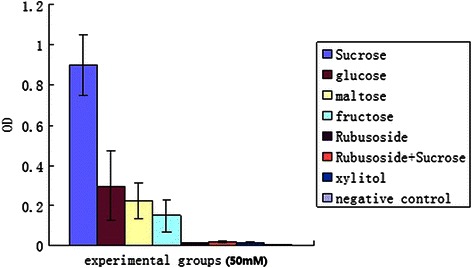
Effects of various experimental groups on the adherence of *S. mutans* to glass surface

The inhibitory effects of the various saccharides on *S. mutans* adherence to a glass surface are summarized in Fig. 3. In the saccharide groups, sucrose-treated *S. mutan*s exhibited the highest adherence to glass, and rubusoside-treated *S. mutan*s exhibited the lowest. When rubusoside was incubated with *S. mutans* in the presence of sucrose, *S. mutans* adherence to glass was increased, but was significantly lower than sucrose (*p* < 0.05) ; and did not significantly differ from that of the negative control.

### Saccharides influence on *S. mutans* acidogenicity

**Fig. 4:**
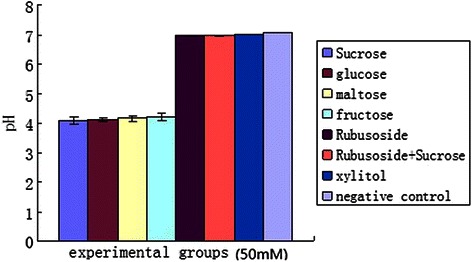
Effects of various experimental groups on the acidogenicity of *S. mutans*

The effects of the various saccharides on *S.mutans* acidogenicity are summarized in Fig. 4. The terminal pH of rubusoside-treated *S. mutan*s was significantly higher than that of the sucrose, glucose, maltose and fructose groups (*p* < 0.05). No significant differences were observed when comparing rubusoside-treated *S. mutan*s to the xylitol group and negative control (*p* > 0.05).

## Discussion

The microbial and dietary factors that cause tooth decay have been studied scientifically for more than 100 years. Frequent and/or excessive sugar (especially sucrose) consumption has been described to play a central role in caries causation, and *S. mutans* appeared to play a key role in metabolizing sucrose to produce lactic acid, which can demineralize enamel [16]. The cariogenic nature of sucrose has led to an intensive search for alternative compounds possessing sweetness without cariogenicity [17]. Sucrose substitutes that are not metabolized by plaque-forming bacteria, particularly *S. mutans*, have been proposed as a promising approach to caries prevention [18].

Rubusoside is a naturally sweet substance isolated from the leaves of the plant *Rubrus suavissimus* S. Lee. (Rosaceae), and was rated as sweeter than sucrose. Sucrose has been established to have the strongest cariogenicity, with glucose, maltose and fructose in decreasing cariogenicity. Xylitol is non-cariogenic and superior to the other sweetener for caries control [19–21]. In the present study, we focused on *S. mutan*s growth, adherence and acid production when incubated with rubusoside, glucose, maltose, fructose, xylitol, and sucrose.

The results of saccharide effects on *S. mutan* growth showed that OD_540_ of rubusoside-treated group was significantly lower than that of the sucrose (positive control), glucose, maltose and fructose groups. Moreover, rubusoside was significantly less acidogenic than glucose, maltose, fructose and sucrose. However, no significant differences were observed when rubusoside was compared to the xylitol group and negative control. These results indicate that rubusoside, like xylitol, either cannot be metabolized or is metabolized very slowly by *S. mutans*.

The ability of *S. mutans* to adhere to and form aggregations as plaque on tooth surfaces is closely related to its ability to cause caries [5, 22–24]. Many studies indicate that the production of water-insoluble glucan from sucrose by extracellular glucosyltransferases (GTF) facilitates the bacterial adherence and plays an important role in the formation of the bacterial aggregates which make up plaque [5, 23, 25]. Our present study shows that of the carbohydrate groups, the highest optical density of *S. mutans* adhering to a glass rod was found for sucrose group (positive control) and the lowest for the rubusoside group. This result suggests that rubusoside markedly reduce *S. mutans*. adhesion to solid surfaces. Thus, we propose that rubusoside is not utilized by GTF of *S. mutans* to produce water-insoluble glucan.

Sucrose is cheap, easily produced from sugar cane or sugar beet, high in calories, and is used as a bulk constituent of foods, a role which no other sweetener would be likely to replace. Although rubusoside is highly sweet, it is low in calories [11]. Thus, the apparent non-cariogenic properties of rubusoside suggest a possible role as an additive to sucrose itself. The results of the present experiments with co-fermentation of rubusoside and sucrose by *S. mutans* reveal *S. mutans* growth and adhesion reduction. Moreover, the acidogenicity of sucrose was markedly decreased by the presence of rubusoside. Therefore, rubusoside may affect the cariogenicity of sucrose when used as sweetening additives.

So far, rubusoside^’^s mechanism of action is still unknown. However, it has been established that structure of rubusoside is a diterpene glycosides consisting mainly of steviol and dextrose (C32H50O13) [12]. From our study we proposed that its possible mechanism of action was that as a five-carbon sugar alcohol, rubusoside cannot be digested by *S. mutans*.when it is transported into the cell, where it probably stays bound to the transport protein. This bond is unbreakable by the usual enzymes so the transport protein is tied up. The transport protein cannot go back out to get more glucose to provide the cell with energy. The reduced number of the transport protein is the process thought to reduce acid production in *S. mutans*.

## Conclusion

In the last years, the multifactorial etiology associated to ecological theory is widely accepted. The literature showed that caries is not a *S. mutans* dependent disease [26]. Hence, further in vitro and clinical studies are needed, particularly for assessment of the role played in the dental biofilm. In short, from the present study, rubusoside may not be used as an energy source by *S. mutans* so that no significant production of acids by these organisms will be followed. It may not act as the substrate for the synthesis of glucan by GTF of *S. mutans*, however it may inhibit glucan synthesis from sucrose. It may have some potential as a non-cariogenic, non-caloric sweetener.

### Ethics

Not applicable.

### Consent to publish

Not applicable.

### Availability of data and materials

All the data supporting our findings is contained within the manuscript.

## Abbreviations

ANOVA: analysis of variance
GTF: glucosyltransferases
OD: optical density
PBS: phosphate-buffered saline
